# Genetic Algorithm to Solve Optimal Sensor Placement for Underwater Vehicle Localization with Range Dependent Noises

**DOI:** 10.3390/s22197205

**Published:** 2022-09-22

**Authors:** Murillo Villa, Bruno Ferreira, Nuno Cruz

**Affiliations:** INESC TEC—Institute for Systems and Computer Engineering, Technology and Science, Faculty of Engineering, University of Porto, Rua Dr. Roberto Frias, 378, 4200-465 Porto, Portugal

**Keywords:** optimal sensor placement, genetic algorithm, underwater vehicle, Fisher information matrix

## Abstract

In source localization problems, the relative geometry between sensors and source will influence the localization performance. The optimum configuration of sensors depends on the measurements used for the source location estimation, how these measurements are affected by noise, the positions of the source, and the criteria used to evaluate the localization performance. This paper addresses the problem of optimum sensor placement in a plane for the localization of an underwater vehicle moving in 3D. We consider sets of sensors that measure the distance to the vehicle and model the measurement noises with distance dependent covariances. We develop a genetic algorithm and analyze both single and multi-objective problems. In the former, we consider as the evaluation metric the arithmetic average along the vehicle trajectory of the maximum eigenvalue of the inverse of the Fisher information matrix. In the latter, we estimate the Pareto front of pairs of common criteria based on the Fisher information matrix and analyze the evolution of the sensor positioning for the different criteria. To validate the algorithm, we initially compare results with a case with a known optimal solution and constant measurement covariances, obtaining deviations from the optimal less than 0.1%. Posterior, we present results for an underwater vehicle performing a lawn-mower maneuver and a spiral descent maneuver. We also present results restricting the allowed positions for the sensors.

## 1. Introduction

Localization is fundamental in the operation of autonomous underwater vehicles (AUVs). These vehicles traditionally rely on inertial and/or acoustic-based relative localization. The latter guarantees bounded error localization but the performances depend on geometrical configurations of the entire system, which is composed of beacons placed on the operation area and on the AUV itself. The usual systems are commonly classified as long baseline (LBL), short baseline (SBL), and ultra-short baseline (USBL), according to the distance between the hydrophones that receive the signals emitted by the AUV. The requirements, such as localization performance, energy consumption, and size, and also the types of measurements used in each system can be very different. LBL sensors are usually hundreds to thousands of meters apart and are commonly placed on buoys on the water surface or in structures mounted on the sea bottom. In an SBL system, the receivers are usually mounted on a ship a few dozen meters apart, while USBL sensors, on the other hand, are usually mounted in a smaller device, being only a few centimeters apart.

The most common measurements used include the time of arrival (ToA), time difference of arrival (TDoA), angle of arrival (AoA), and the received signal strength (RSS). With bigger baselines, the usual approach is to use the ToA of the received signal at hydrophones, and with the propagation speed of sound, the range of the sensor to the target can be obtained. Trilateration can be performed by supplying the range measurements to an elliptical positing algorithm to estimate the vehicle’s position. These systems require synchronization between emitters and receivers or a technique that allows estimating the time of the emitted signal. With smaller baselines, the receivers are more susceptible to signal noises, and usually, the time difference of arrival between the receivers is used. These systems are more easily deployed, however, synchronization offsets between receivers, as well as the deviation in the estimation of the receivers’ positions, can highly degrade the localization performance [1]. The angle of arrival systems uses the angles at which the signal arrives at the sensors to estimate the target position using triangulation. On the other hand, RSS systems rely on path loss propagation models and the measurement of the power of the received signal to estimate the range between the sensor and target.

Regardless of the localization system, the geometry between sensors and target can strongly affect the performance of the localization algorithm [2], whereas an inadequate sensor placement can lead to large localization errors [3]. Therefore, the sensor placement should be done to maximize the system performance at localizing the vehicle. This work will address optimum sensor placement for improving the localization of underwater vehicles. The remainder of this introduction will present a brief summary of the literature on this topic focusing on some key aspects: measurements considered; measurement noise models; evaluation criteria; optimization methods. After, we present a comparison table of our work with some relevant work in the literature.

The literature has extensively analyzed the problem of optimal sensor placement for source localization. Regarding the measurements between sensors and source, most works have considered direct range [2,3,4,5,6,7,8], time of arrival [2,5,9], difference time of arrival [10,11,12,13,14,15,16], angle of arrival [2,5,17], or received signal strength [18]. However, although in various applications the measurement noises usually varies with distance, especially in underwater scenarios [19], the most common assumption is to consider noise to be independent of the distance and equal to all the sensors. Some exceptions include the work of Yang et al. [16], which considers TDoA measurements and presents a complete model of the noise depending on inter-distances, as well as a model of the synchronization offsets and the sensor location errors. In [6,19], the measurements are ranges, and the noise is a Gaussian process in which the variance depends on distance. In the work by Bo et al. [18], the measurements are the RSS, and the noise is added by a factor that depends on the distance between the emitter and receiver. The sound speed profile (SSP) on the water is considered, and the sound velocity is modeled as a linear function of depth. Domingo-Perez et al. [20] consider the range differences between the sensors as the measurements and the noise is considered inversely proportional to the square of the distance between the emitter and receiver.

A common approach to evaluating the system’s performance is by analyzing the Fisher information matrix (FIM) and the Cramer–Rao lower bound (CRLB). The FIM captures the amount of information that measured data provides about an unknown parameter, while the CRLB is the lower bound for the covariance matrix of any unbiased estimator. Under known assumptions, the CRLB is the inverse of the FIM. In this regard, it is possible to derive scalars from the FIM that can be used to evaluate the performance of a particular solution. The most common criteria for optimality is the determinant of the FIM [2,3,4,5,6,7,8,10,11,12,14,16,17,18], although the trace of the inverse of the FIM [9,15,17], the eigenvalues of the inverse of the FIM or a combination of them are also found in the literature [20]. In the optimal experimental design theory, the strategies that optimize based on these three criteria are classified as D-optimality, A-optimality, and E-optimality, respectively. Additionally, a geometric interpretation exists for each of these criteria. For example, the D-optimum criterion minimizes the volume of the uncertainty ellipsoid, the A-optimum criterion minimizes the average variance of the estimate, and the E-optimum criterion minimizes the length of the most significant axis of the uncertainty ellipsoid. Recently, the work by Sahu et al. [21] proposes a framework to combine the three above criteria under a general approach for problems of optimal sensor placement for localizing stationary targets.

Although less common, it is possible to find optimization criteria that are not FIM based. One example is the work done by Neering et al. [13], which approaches the problem with a procedure that aims to minimize the condition number of an analytic linear least-squares (LLS) estimator and an iterative, linearized model (LM) estimator. Another example is the work by Levanon [22], which uses the geometric dilution of precision (GDOP) as the optimization criteria. GDOP is a metric initially developed for selecting optimal 3D geometry of satellites in global positioning systems (GPS) and is a metric that describes the effect of geometry on the relationship between the measurement and position error [23]. Usually, the lower the GDOP, the better the geometric configuration [24]. Du et al. [25] uses the area of the ellipse of uncertainty of an iterative estimation filter to optimize the formation of AUVs.

Usually, the problem of optimal sensor placement is solved considering a single optimization criterion. However, the work done by Domingo-Perez [20] considers a multi-objective optimization problem, where the objective is to find the optimum solution considering combinations of the three criteria mentioned above, in pairs, as well as the ratio between the eigenvalues of the inverse of the FIM and the uncertainty in a predefined direction. As the objectives can conflict, the final solution requires a trade-off between objectives. In this sense, the author finds the Pareto front for these criteria pairs, which represents the set of solutions whose objectives cannot be improved without deteriorating at least one of the others. Multi-objective optimization is also used in the context of multi-object tracking, for example in [26], where the authors estimated the Pareto front considering the determinant of the FIM as a metric. With this approach, the authors could analyze the trade-offs in localization performance of the different targets using different sensor placements. Additionally, in the context of multi-target tracking, the authors of [27] propose a different approach that uses shared sensors and combines analytical solutions with a numerical algorithm to optimize the positioning of sensors according to the A-optimality criterion.

The problem of sensor placement for localization has been assigned as NP-hard, making it computationally intractable to evaluate all possible configurations [23,28,29]. Concerning the solution method, in the context of localization systems for underwater vehicles, the most common approach is to derive optimal conditions for specific cases and find solutions that meet these conditions. In this sense, the work done by Bishop et al. [2,4,10] and Moreno-Salinas [3,8,30] are relevant. In the former, the authors present results for bearing, range, and ToA and TDoA measurements in two-dimensional problems. In the latter, the authors find solutions considering bearing and range measurements in two and three-dimensional scenarios. For range measurements in 3D, solutions are also found by restricting the sensors to rely on a plane and optimizing for a region of uncertainty instead of the common approach of considering a known target position. Another work that became a recurrent reference is the one of Martinez and Bullo [7], where they proposed a positioning algorithm for moving sensors that maintain an optimal geometry while tracking a target. In our previous work [31], four autonomous surface vehicles in formation were employed to track an underwater unsynchronized target from ToA measurements. The geometric configuration of the formation was based on the D-optimality criterion.

With the increasing complexity of the problem, mathematical derivations of optimal solutions became harder. In some contexts outside the scope of marine applications, the sensor placement problem for localization is known as the node location problem (NLP), and it is commonly addressed for local positioning systems (LPS) that do not rely on GPS information. In these contexts, metaheuristics methods are more commonly applied. Ferrero-Guillén et al. [29] presents an analysis of GAs selection and crossover techniques for node location problems using the square root of the trace of the inverse FIM as a fitness function. The authors compared tournaments 2 and 3 (T2, T3) and roulette (R) and ranked roulette (RR) selection techniques and single, two, and three points (1P, 2P, and 3P) along with uniform crossover techniques. The authors obtained the best results for a combination of T2 with 1P crossover. However, the authors implemented and concluded that a hybrid technique, named hybrid GA, with two stages, first with T3 and 3P crossover followed by a refined search with T2 and 1P, could surpass any individual combination. In [32], Díez-González et al. proposed a GA considering two scenarios with different constraints for the position of the target. The authors used five sensors, TDoA measurements with the root of the inverse FIM as a fitness function, along with a T3 selection and 1P crossover strategies. They observed that independently of the initial random population, each of the five sensors converged to a specific region of the grid map, which was posteriorly used for grid refinement. In a posterior work, Díez-Gonzalez et al. [28] proposed a combination of a hybrid GA with a memetic algorithm that increased the accuracy by 14.2% with regards to the previous works. More recently, in [33], the authors further extended their work to consider failure conditions of the sensors and estimate the positioning that guarantees the convergence of their localization algorithm.

### 1.1. Novelty

To highlight the novelty of our work, we present in Table 1 the differences between our work and some of the relevant literature. We focus on works that had the most similar optimization objectives to ours to emphasize the differences in similar works. Therefore, we only present works in which the objective was to optimize a metric based on the FIM. Moreover, we classified the literature according to the following parameters:

1. Optimization method
  (a) Analytical (Analy)
  (b) Heuristics and Meta-heuristics (HM)
2. Optimization criteria
  (a) Determinant of the Fisher information matrix (D)
  (b) Trace of the inverse of the Fisher information matrix (A)
  (c) Eigenvalue of the inverse of the Fisher information matrix (E)
  (d) Multi-objective (Multi)
3. Measurement noise considerations
  (a) Parameter independent (PI)
  (b) Parameter dependent (PD)
4. Measurements
  (a) Range (R)
  (b) Range difference (RD)
  (c) Time of arrival (ToA)
  (d) Difference time of arrival (TDoA)
  (e) Angle of arrival (AoA)

Our work applies a genetic algorithm (GA) to address the optimization of sensor locations to localize an underwater vehicle by minimizing the average maximum eigenvalue of the inverse of the FIM along the vehicle’s trajectory. From Table 1 it is possible to observe that the majority of the literature disregards the E-optimality criterion in sensor optimization problems, probably due to the difficulty in deriving a closed expression for the computation of the eigenvalues of the inverse of the FIM. The work of Domingo-Pérez et al. [20] is, as ours, one of the few that considers this criterion for the evaluation of sensor placement. However, we differ from [20] as we consider a different measurement technology and present results for a set of scenarios typical in autonomous underwater vehicle operations. Moreover, our proposed genetic algorithm is modified to contain a crossover technique that propagates characteristics of the population-specific problem of sensor placement, which to the best of the author’s knowledge has never been done before.

Results are initially compared with known optimal solutions and posteriorly presented for a lawn-mower and spiral descent maneuver considering configurations from four to eight sensors in a plane. In the lawn-mower case, we also present results restricting the search space to half of the initial plane. Although the method present can be used for practically any trajectory of the vehicle, we chose these two maneuvers to perform the simulations, as they are among the most traditional for autonomous underwater vehicles. The lawn-mower is usually used in mapping operations [34,35], while the spiral descent is typical of AUVs with flat wings for descending in the water column [36] or to cover an area during the descent [37].

### 1.2. Major Contributions

In the present work, the problem of optimum sensor placement is addressed in the scope of target localization with range-only measurements. By applying a genetic algorithm (GA), one of this paper’s contributions relies on presenting a method that can be used as a planning tool for missions that require localizing underwater vehicles considering more realistic and complex scenarios. For example, one can use the presented algorithm beforehand to find the sensor placement that maximizes the information about the target during a mission. The proposed GA can avoid the need for specific scenarios, and solutions can be found for many cases. Unfortunately, this type of strategy has been chiefly disregarded in the marine applications literature. Additionally, the results for a relatively complex scenario employing a range-dependent model of measurement noise and metrics considering the entire planned trajectory are presented and discussed. To the best of our knowledge, this paper is the first approach to be presented for finding the optimal solutions combining these two features for underwater localization. Additionally, the power of the GA is further exploited by considering also a multi-objective problem, considering two pairs of criteria: the average maximum eigenvalue of the inverse of FIM and the average determinant of FIM, and the average maximum eigenvalue and the average trace of the inverse of FIM. The Pareto fronts are presented for these pairs of criteria for the lawn-mower maneuver and four sensors in a plane.

The remainder of this paper is organized as follows: Section 2 formalizes the problem description and details the evaluation metrics used. Following, Section 3 describes the developed GA. In Section 4, we present the simulations and discuss the results. Finally, the last section presents the conclusions.

## 2. Measurement Model and Problem Description

We consider the problem of sensor placement in $R^{3}$ for target localization in $R^{3}$. Let $q={\left[\begin{array}{ccc} q_{x} & q_{y} & q_{z} \end{array}\right]}^{T}$∈$R^{3}$ denote each position of a target in a grid of points. Let $p_{i}\in R^{3}$, i∈{1,2,3…,n} denote the positions of *n* sensors on the same grid of points. The sensors will be restricted to lie on plane, constraining its coordinates to $p_{i}={\left[\begin{array}{ccc} p_{xi} & p_{yi} & 0 \end{array}\right]}^{T}$. Now, let us consider the measurements of each sensor and the actual distance between sensor and target corrupted by some additive noise. Then, the measurement between the *i*th sensor and the target is given by

(1) $$z_{i}=\left|q-p_{i}\right|+\omega_{i}=r_{i}+\omega_{i}\;,$$

where the operator | · | denotes the Euclidean norm, $r_{i}$ is the distance between the *i*th sensor and the target, and $\omega_{i}$ is the additive measurement noise.

The distance measurements will be affected by effects such as multi-path, the variability of the speed of sound in the water column, and the degradation of the signal-to-noise ratio (SNR), among others. It is reasonable to infer that the quality of the measurements will decay with the increase in distance between the sensors and the target. Therefore, we modeled the measurement noise $\omega_{i}$ as a Gaussian process in which the variance depends on the distance. The same model was used by [6,19,38] and is mathematically stated as:

(2) $$\omega_{i}=\left(1+\eta r_{i}\right)\omega_{0},$$

where $\omega_{0}$ is the uncorrelated white Gaussian noise, i.e., $\omega_{0}∼N\left(\mu_{0},\;\sigma_{0}^{2}\right)$ , η is a scalar, and $\omega_{0}\eta r_{i}$ models the distance-dependent term. The expected value and the variance of each measurement are then given by

(3) $$\begin{array}{cc} E(z_{i}) & =r_{i}+\mu_{0}\left(1+\eta r_{i}\right)\;, \end{array}$$

(4) $$\begin{array}{cc} Var(z_{i}) & =\sigma_{0}^{2}{\left(1+\eta r_{i}\right)}^{2}\;. \end{array}$$

Note that the expected values of the measurements are the true value of the distances $r_{i}$ added by a bias term $\mu_{0}\left(1+\eta r_{i}\right)$ that depends on the true distance and the mean value of $\omega_{0}$. If the additive noise has zero mean, the measurements are unbiased. For generalization, the derivations in the following sections make no assumptions on the mean value of the additive noise term. However it is important to state that it is assumed the existence of unbiased estimators able to estimate the target’s positions with measurements as presented above. This may be unfeasible with biased measurements and the derivation of such estimators is beyond the scope of this work. Nevertheless, the results presented considered the term $\mu_{0}$ as zero.

We want to select the best sensors locations $p_{i}$ in a plane, with respect to some well-defined criteria:

1. the range measurement model of each sensor;
2. a 3D grid of points;
3. the positions *q* that define the trajectory of a target in the 3D grid of points.

### 2.1. Evaluation Criteria

The CRLB is the minimum variance that unbiased estimators can achieve. It is the optimal mean square error for any unbiased estimator [39]. It does not say anything about the existence of such unbiased estimators, but it gives us a metric for performance comparison. For unbiased estimators, the CRLB is given by the reciprocal of the Fisher information or the inverse of the FIM for vector cases.

Let $q\in R^{3}$ be the target position, $\hat{q}$ an estimate of *q* obtained by the measurement vector *z*, and L(z|q) the likelihood function for *z*. Then, the variances of the coordinate estimates are bounded by

(5) $$Var\left(\hat{q_{k}}\right)\geq {\left[J{\left(q\right)}^{-1}\right]}_{{kk}^{\prime }}$$

where J(q) is the 3×3 FIM, defined by:

(6) $${\left[J{\left(q\right)}^{-1}\right]}_{kj}=-E\left[\frac{\partial^{2}\ln L(z|q)}{\partial q_{k}\partial q_{j}}\right]\;,$$

and k∈{1,2,3} and j∈{1,2,3} are the indexes representing the Cartesian coordinates of the target and positions of the FIM.

Now, let us consider *n* sensors under the assumption of additive Gaussian measurement noise,

(7) z∼N(μ(q),Σ(q)) .

where $\mu \left(q\right)\in R^{n}$ is the mean vector and $\Sigma \left(q\right)\in R^{n\times n}$ is the covariance matrix. The likelihood function of *z* is:

(8) $$L\left(z|q\right)=\frac{1}{{\left(2\pi \right)}^{\frac{1}{n}}{\left|\Sigma \left(q\right)\right|}^{\frac{1}{2}}}\exp\left[-\frac{1}{2}{\left(z-\mu \left(q\right)\right)}^{T}\Sigma^{-1}\left(q\right)\left(z-\mu \left(q\right)\right)\right]\;.$$

A general expression for the FIM can be derived for the Gaussian case with the mean and covariance depending on the parameter to be estimated. For a complete derivation of the expression, the reader can refer to [40]. Following this expression, the FIM can be computed as

(9) $$J_{kj}=\frac{\partial \mu^{T}\left(q\right)}{\partial q_{k}}\Sigma^{-1}\left(q\right)\frac{\partial \mu (q)}{\partial q_{j}}+\frac{1}{2}tr\left(\Sigma^{-1}\left(q\right)\frac{\partial \Sigma (q)}{\partial q_{k}}\Sigma^{-1}\left(q\right)\frac{\partial \Sigma (q)}{\partial q_{j}}\right)\;.$$

From Equations (3) and (4), the mean and covariance of the measurement vector are given as:

(10) $$\begin{array}{c} \mu \left(q\right)={\left[r_{1}+\left(1+\eta r_{1}\right)\mu_{0},\ldots ,r_{n}+\left(1+\eta r_{n}\right)\mu_{0}\right]}^{T} \end{array}$$

(11) $$\begin{array}{c} \Sigma \left(q\right)=\left[\begin{array}{ccc} \sigma_{0}^{2}{\left(1+\eta r_{1}\right)}^{2} & 0 & 0\\ 0 & \ddots & 0\\ 0 & 0 & \sigma_{0}^{2}{\left(1+\eta r_{n}\right)}^{2} \end{array}\right] \end{array}$$

Following are the derivatives of the mean vector and the covariance matrix with respect to the target positions:

(12) $$\begin{array}{c} \frac{\partial \mu_{i}\left(q\right)}{\partial q_{x}}=\left(1+\mu_{0}\eta \right)\left(\frac{q_{x}-p_{xi}}{r_{i}}\right) \end{array}$$

(13) $$\begin{array}{c} \frac{\partial \mu_{i}\left(q\right)}{\partial q_{y}}=\left(1+\mu_{0}\eta \right)\left(\frac{q_{y}-p_{yi}}{r_{i}}\right) \end{array}$$

(14) $$\begin{array}{c} \frac{\partial \mu_{i}\left(q\right)}{\partial q_{z}}=\left(1+\mu_{0}\eta \right)\left(\frac{q_{z}-p_{zi}}{r_{i}}\right) \end{array}$$

(15) $$\begin{array}{c} \frac{\partial \Sigma_{ii}\left(q\right)}{\partial q_{x}}=2\eta \sigma_{0}^{2}\left(1+\eta r_{i}\right)\left(\frac{q_{x}-p_{xi}}{r_{i}}\right) \end{array}$$

(16) $$\begin{array}{c} \frac{\partial \Sigma_{ii}\left(q\right)}{\partial q_{y}}=2\eta \sigma_{0}^{2}\left(1+\eta r_{i}\right)\left(\frac{q_{y}-p_{yi}}{r_{i}}\right) \end{array}$$

(17) $$\begin{array}{c} \frac{\partial \Sigma_{ii}\left(q\right)}{\partial q_{z}}=2\eta \sigma_{0}^{2}\left(1+\eta r_{i}\right)\left(\frac{q_{z}-p_{zi}}{r_{i}}\right) \end{array}$$

Substituting Equations (12) to (17) into Equation (9), the following expression for the FIM is obtained:

(18) $$J=\Theta \sum_{i=1}^{n}\frac{1}{{\left(1+\eta r_{i}\right)}^{2}}\tau_{i}\tau_{i}^{T}\;,$$

where $\tau \in R^{3}$ is the vector defined as:

(19) $$\tau_{i}=\left[\begin{array}{c} \frac{q_{x}-p_{xi}}{r_{i}}\\ \frac{q_{y}-p_{yi}}{r_{i}}\\ \frac{q_{z}-p_{zi}}{r_{i}}\;\; \end{array}\right]\;,$$

and Θ is a constant term given by:

(20) $$\Theta =\sigma_{0}^{-2}{\left(1+\eta \mu_{0}\right)}^{2}+2\eta^{2}\;.$$

By exploiting the potential of genetic algorithms over metrics based on the FIM, solutions can be found considering both a single-objective and a multi-objective problem. In the multi-objective case, we estimated the Pareto front and analyzed how the sensor topology evolved along the solutions in the front.

### 2.2. Single-Objective Optimization

Common optimization objectives are to minimize the determinant, trace, or maximum eigenvalue of the inverse of the FIM. When considering a single-point target, the first gives a scaled measure of the volume of the uncertainty ellipsoid of the estimation, the second the sum of the squared length of the axes of the uncertainty ellipsoid axis, and the last the maximum length among all axes.

If minimizing based on the determinant (volume of the uncertainty ellipsoid) or the trace (average of uncertainty ellipsoid axis), configurations of sensors that give very low uncertainty in a particular direction, but very high in other are possible solutions. To converge to configurations that have small uncertainties in all directions, we opted for minimizing the maximum eigenvalues of the inverse of the FIM. Therefore, we are focusing on minimizing the worst axis of uncertainty and avoiding solutions that could have high uncertainties in a particular direction.

To consider cases in which the target performed a path, the metrics had to be aggregated over all possible target positions. The choice of the aggregation function defines a unique objective to be optimized [5]. Following the formulation presented in [5], consider $x_{1},x_{2},\ldots ,x_{n}$ as *n* arbitrary positive numbers, then a generalized mean can be defined as:

(21) $$M_{r}\left(x_{1},x_{2},\ldots ,x_{n}\right)={\left(\frac{1}{n}\sum_{j=1}^{n}x_{j}^{r}\right)}^{\frac{1}{r}}\;\;,$$

where *r* controls the type of aggregation function. Common functions are the min, max, geometric, harmonic, and arithmetic mean, corresponding to r=−∞, r=∞, r=0, r=−1, and r=1, respectively. For fixed $x_{1},x_{2},\ldots ,x_{n}$, these functions are monotonically increasing in *r*, and bounded such that

(22) $$\begin{array}{cc} \; & min\left(x_{1},x_{2},\ldots ,x_{n}\right)\leq M_{r}\left(x_{1},x_{2},\ldots ,x_{n}\right)\\ \; & \leq max(x_{1},x_{2},\ldots ,x_{n})\;\mathrm{for}\;-\infty \leq r\leq \infty \end{array}$$

In this work, the evaluation metric considered is the arithmetic average of the maximum eigenvalue of the inverse of the FIM along the vehicle path. The adoption of the arithmetic average instead of any other type of average reflects the fact that we consider all target positions to be equally important in the decision of sensor placement.

### 2.3. Multi-Objective Optimization

In multi-objective optimization, one wants to minimize or maximize multiple objective functions subject to a set of constraints. Let *f* be a vector function that maps *m* parameters, or decision variables, to a tuple of *n* objectives. Formally, one wants to:

$$\begin{array}{cc} \min/\max\;\; & y=f\left(x\right)=\left(f_{1}\left(x\right),f_{2}\left(x\right),\ldots ,f_{n}\left(x\right)\right)\\ \mathrm{subject}\;\mathrm{to}\;\; & x=(x_{1},x_{2},\ldots ,x_{m})\in X\\ \; & y=(y_{1},y_{2},\ldots ,y_{n})\;\in Y\\ \; & g_{j}\left(x\right)\leq 0,\;j=1,2,\ldots ,J\\ \; & h_{k}\left(x\right)=0,\;k=1,2,\ldots ,K \end{array}$$

where *x* is the decision vector, *X* is the parameter space, *y* is the objective vector, *Y* is the objective space, and $g_{j}$ and $h_{k}$ are constraints.

The objectives can conflict, meaning the final solution requires a trade-off between objectives. According to Konak, Coit, and Smith [41], there are two general approaches for multi-objective optimization. The first combines the multiple objectives into a single function or treats all but one objective as a constraint. A proper selection of a utility function to combine the multiple objectives can be very difficult, as scaling of the objectives is needed and small perturbations on weights can lead to very different solutions. Treating the objectives as constraints can be rather arbitrary, and therefore the optimum solution.

The second general method focuses on estimating the set of Pareto optimum solutions. The Pareto set, also commonly referred to as the Pareto front or Pareto frontier, consists of solutions in which it is impossible to improve one objective without deteriorating at least one of the others. These are called non-dominated solutions. Following [42], let us assume, without loss of generalization, a maximization problem. Formally, a point $x^{0}\in X$ is said to be Pareto optimal with respect to an objective vector if there does not exist x∈X in which the following are true:

$$\forall \;k\in \left\{1,2,\ldots ,n\right\}:f_{k}\left(x\right)\geq f_{k}\left(x^{0}\right)$$

and

$$\exists \;l\in \left\{1,2,\ldots ,n\right\}:f_{l}\left(x\right)>f_{l}\left(x^{0}\right)$$

In this case, we say that the point $x^{0}$ dominates all other points x∈X.

Two pairs of criteria were considered when estimating the Pareto front. The first considered minimizing the maximum eigenvalue and the trace of the inverse of the FIM, while the second minimizing the maximum eigenvalue and the determinant of the FIM.

## 3. Genetic Algorithm for Sensor Placement

Genetic algorithms are inspired by evolutionary theory, where species that do not adapt to the environment are faced with extinction by natural selection. The individuals that better fit the conditions survive and have greater opportunities to generate offspring and pass their genes. With the passing generations, the individuals carrying the correct combination of genes will be dominant. It is also possible that random changes may occur in some individual genes. These mutations can generate additional advantages to survival. Unsuccessful changes are eliminated by natural selection.

**Figure 1:**
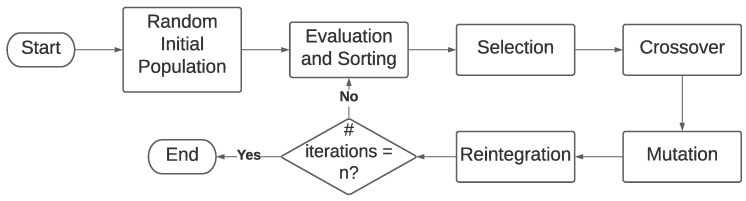
Genetic algorithm diagram.

We present the general workflow of the implemented GA in Figure 1.

**Figure 2:**
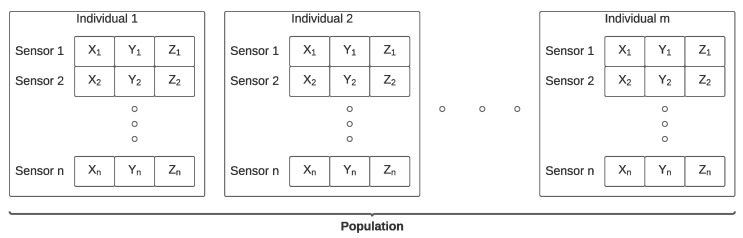
Representation of a population.

The first step is the generation of an initial random population of size *s*. Each individual of the population is a possible solution to the problem and consists of the Cartesian coordinates of each of the sensors. Figure 2 presents a representation of a population.

Next, we evaluate the entire population according to the appropriate fitness function and sort the individuals from best to worst. We use the sorted population to substitute the less fitted individuals with new ones at the end of each iteration. The genetic algorithm is applied to both single and multi-objective problems, where the major difference relies on the evaluation function. In the single-objective case, we want to minimize the arithmetic mean of the maximum eigenvalue of the inverse of the FIM along the vehicle trajectory. However, in the multi-objective case, when estimating the Pareto front for pairs of optimization criteria, it is unreasonable to sort the population considering just one of the criteria. Therefore, in this work, we use the fast nondominated sorting with a crowd comparison approach as defined in the NSGA-II algorithm [43]. First, we create the nondomination rank, where first are all solutions that are not dominated by any other solutions. In our case of two optimization criteria, for a solution to be non-dominated by any other means that does not exist any solution that is better than the first considering both the criteria. For example, if solution A is better than solution B considering both criteria (minimum/maximum eigenvalue of the inverse of the FIM and the maximum determinant of the FIM, for example), then solution A dominates solution B, and solution B could not be attributed a first position in the nondomination rank. However, if solution C is better than solution A in one parameter but worst in the other, and there is no other solution that is better than these when considering both parameters, then solution A and C would both receive the first position in the nondomination rank. The solution with the first positions in the nondomination rank are the solutions of the first front. The subsequent positions in the nondomination rank are constructed excluding the solutions on a better rank and following the same procedure used to construct the previous rank positions. This nondomination rank is the first criterion for sorting. To guarantee a good spread of solutions, crowd distance is used as a second sorting criterion. To calculate the crowd distance, the solutions are sorted in ascending order for each optimization criterion. Afterward, the best and worst solutions for each objective function are attributed an infinity distance. The remaining intermediate solutions are attributed a distance equal to the absolute normalized difference in the function values of two adjacent solutions. Afterward, for solutions in the same nondomination rank, we prioritize the one with a bigger crowd distance.

Following, the selection phase selects the individuals who generate offspring in the crossover phase. We randomly select $\alpha_{P}$% members of the population as contestants in a tournament, where $0<\alpha_{p}\leq 100$ is a predefined parameter. All contestants generate offspring. However, we evaluate them, and the crossover is made by taking the most fitted ones two by two. That means that the most fitted contestants will generate offspring with one another. Two offspring are generated for each pair of parents. One characteristic of each parent is selected to propagate to the offspring. To preserve information about the spread of sensors around the target, these characteristics are the distance and direction of each sensor from the center of the plane where the sensors are positioned. As we can always translate our map to the center of the path of the AUV around the center of the plane, the range and direction could give a good perspective of the distribution of the sensors around the vehicle’s path. Therefore, for the first child, we maintain the direction of the first parent for each sensor, but use the distances of the second parent. For the second child, we maintain the distances of the first parent, but use the directions of the second parent.

After the generation of offspring, the mutate phase randomly selects $\alpha_{M}$% of individuals in the solution to have a mutation. The mutation is made by randomly choosing a sensor for each selected individual and by moving these sensors fifty units in a random direction. This phase will increase the diversity, avoiding the convergence to a homogeneous population that potentially makes it harder for the next generations to improve fitness. Finally, the last phase is reintegration, where we reinsert the offspring generated in the crossover and the mutated individuals in the solution. Finally, we reinsert by substituting the less fitted individuals in the solution with the new ones. The algorithm stops after a predefined number of iterations and the individual with the best performance is selected as the final solution.

## 4. Simulations and Results

This section initially presents the simulation settings common to all cases and after presents each case’s results.

### 4.1. Simulation Setup

In all cases, the sensors were restricted to a plane in the Z=0 coordinate. Therefore, the search was performed in a 2D grid of points of size 3000×3000. It is assumed that the step on the grid is of 1 m. The size of the grid combined with the step size of 1 m makes the search space compatible with most real underwater vehicles’ operational area. Moreover, 1 m is compatible with the accuracy of global navigation satellite systems, which are traditionally used to estimate the position of the sensors at the water surface. In all simulations, the measurement noise parameters were $\mu_{0}=\;$0 m, $\sigma_{0}^{2}=\;$ 0.5 $m^{2}$, and η= 0.01 m m^−1^.

For the GA, we chose an $\alpha_{p}=20\%$ for the tournament between individuals and $\alpha_{M}=20\%$ for the mutation. The simulations were made for a population of 500 individuals during 2000 iterations. The parameters of the GA were chosen based on preliminary simulations where the chosen parameters presented the best results while maintaining relatively low computational times. All simulations used four cores of an Intel i7-8550U 1.8 GHz CPU. In all simulated cases, we present the computational time; however, the focus of this work is not on the computational burden of the algorithm but on the analysis of the sensor configurations found. Nevertheless, we present them to the interested reader.

### 4.2. Comparison with Known Optimal Solution

Simulations for a case with a known optimal solution were made to validate the algorithm’s performance. The scenario is a point target with the sensors restricted to lie on a plane and measurement noise independent of range and equal to all sensors. Thus, in this case, η was set to zero. This case was evaluated in [3] and has a known optimum FIM given by

(23) $$J=\frac{1}{\sigma^{2}}\left[\begin{array}{ccc} \frac{n}{3} & 0 & 0\\ 0 & \frac{n}{3} & 0\\ 0 & 0 & \frac{n}{3} \end{array}\right]\;\;,$$

where *n* is the number of sensors and σ is the constant and equal to all sensors’ measurement noise. Therefore, all three eigenvalues of the inverse of the FIM are equal to $\frac{3\sigma^{2}}{n}$.

The cases with four, five, six, seven, and eight sensors were simulated ten times each. Table 2 presents the mean results for 10 simulations in each case.

For this case, optimal solutions are found by positioning the sensors equally distributed in a circle with the target projection at the center of the circle. The optimum radius of the circle is given by $z_{t}\sqrt{2}$, where $z_{t}$ is the target depth. We simulated the target at a depth of 500 m, giving an optimum radius of approximately 707 m. Although the algorithm did not find any optimal solution, all results presented deviations less than 0.1% from the optimal. The discretization of the grid also partially contributed to deviations from the optimal.

### 4.3. Sensors in a Plane with Lawn-Mower

In this set of simulations, the vehicle performed the lawn-mower maneuver at a constant depth, at Z=900, and the total area covered by the vehicle was 400,000 m${}^{2}$. Simulations were performed for configurations of four, five, six, seven, and eight sensors. We use the case with four sensors to present an in-depth analysis and present the results of the remaining cases in the form of tables.

**Figure 3:**
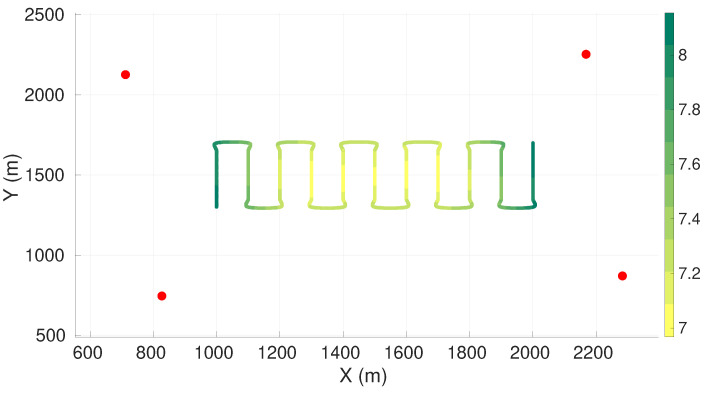
Maximum axis of uncertainty along vehicle trajectory for lawn-mower maneuver and four sensors. Red dots represent the sensors, and the green gradient line represents the maximum axis of uncertainty along the vehicle trajectory.

Figure 3 presents the value of the maximum axis of uncertainty (the square root of the maximum eigenvalue) along the vehicle trajectory for the best found configuration of four sensors. Table 3 presents the respective sensor positions. The sensor positions for the cases with five, six, seven, and eight sensors are presented in Appendix A.1.

From Figure 3 we observe that the worst uncertainty is at the beginning and the end of the trajectory. To enable a better understanding of the values of the worst axis of uncertainty, we computed the standard deviation, $\sigma_{c}$, of the range measurements of each sensor to the center of the target plane (coordinates x=1500, y=1500, z=900), according to Equation (2). These parameters are presented in Table 4.

In all cases, the sensors were approximately at an equal distance from the center of the target plane and approximately equally distributed around the center of the plane. Therefore, we only present the mean distance to the center and the mean standard deviation for the range measurements for the remaining cases.

Table 5 presents the worst axis of uncertainty along the entire vehicle trajectory, the mean standard deviation $\sigma_{c}$ for the range measurement to the center of the target plane, and the computational times in seconds.

Due to how the Fisher information matrix is constructed, increasing the number of sensors should always increase the information, or decrease the uncertainty of the estimation. Therefore it is expected that the worst axis of uncertainty decreases with the increase in the number of sensors, which was observed in all the simulated cases.

### 4.4. Sensors Restricted to Half of the Plane with Lawn-Mower

**Figure 4:**
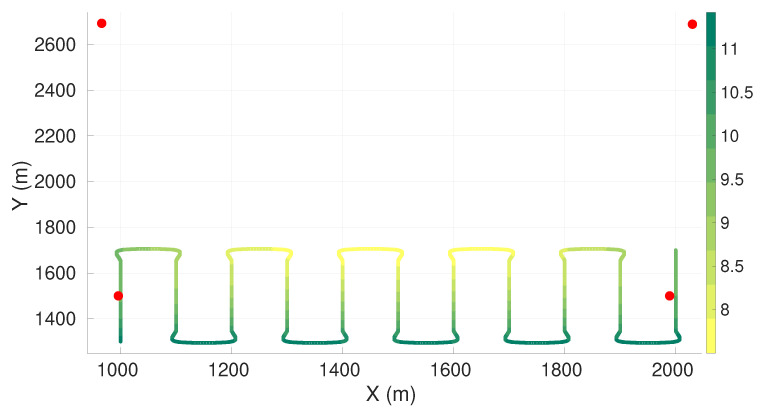
Maximum axis of uncertainty along vehicle trajectory for the lawn-mower maneuver and four sensors. Red dots represent the sensors, and the green gradient line represents the maximum axis of uncertainty along the vehicle trajectory.

In this set of simulations, we restrained the sensors positioning to half of the original plane. Figure 4 presents the maximum axis of uncertainty along the vehicle trajectory for the four sensors case. Table 6 presents the respective sensor positions. The sensor positions for the cases with five, six, seven, and eight sensors are presented in Appendix A.2.

Comparatively to the entire plane case, it is possible to see that all the sensors tended to come closer to the vehicle trajectory in the x-direction. We observed a decrease in localization performance compared to the unrestricted case. Additionally, there is a transition of the worst axis of uncertainty to the bottom part of the trajectory, where sensors were not allowed. Additionally, as a consequence of the restriction to half of the plane, there was a shift in the uncertainty along the Y direction.

Table 7 presents the worst axis of uncertainty along the entire vehicle trajectory, the mean standard deviation $\sigma_{c}$ for the range measurement to the center of the target plane, and the computational times in seconds for simulations with four, five, six, seven, and eight sensors.

### 4.5. Sensors in a Plane with Spiral Descent

In the spiral descent maneuver, the vehicle went from depth Z=20 to Z=900 in a circular motion with a radius equal to 100 m. Simulations were performed for configurations of four, five, six, seven, and eight sensors.

**Figure 5:**
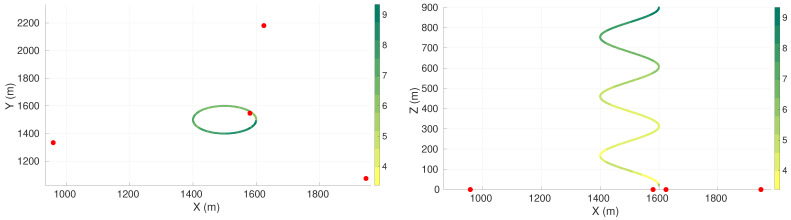
Maximum axis of uncertainty along vehicle trajectory for the spiral descent maneuver and four sensors. Red dots represent the sensors, and the green gradient line represents the maximum axis of uncertainty along the vehicle trajectory.

Figure 5 presents the value of the maximum axis of uncertainty along the vehicle trajectory for the best found configuration of four sensors. The first sub-figure presents the positioning of the sensors in the X–Y plane or a view from the top. The second sub-figure presents a view of the X–Z plane or a view from the side. Table 8 presents the respective sensor positions. The sensor positions for the cases with five, six, seven, and eight sensors are presented in Appendix A.3.

It is possible to observe a high value in the maximum axis of uncertainty, followed by an oscillation in the uncertainty until about the depth of 200 m. After 200 m, the maximum axis of uncertainty tended to increase with depth. Additionally, in all cases it was possible to observe that at least one sensor was very close to the start point of the vehicle trajectory.

For the four-sensor case, the closest sensor to the initial point of the trajectory, at Z=20 m, is at a distance of 95 m of this point, giving a standard deviation of the range measurement of 1.38 m. The most distant sensor to the end of the trajectory, at Z=900 m, is at 1135 m, giving a standard deviation on the range measurement of 8.73 m.

Table 9 presents the worst axis of uncertainty along the vehicle trajectory with the respective computational times in seconds. The increased computational time compared to the lawn-mower maneuver is due to the increased length of the path of the vehicle.

### 4.6. Pareto Front for Lawn-Mower Maneuver

**Figure 6:**
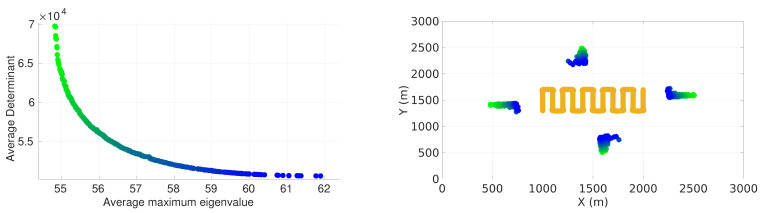
Pareto front considering average maximum eigenvalue and determinant of inverse of FIM with respective positioning of sensors.

**Figure 7:**
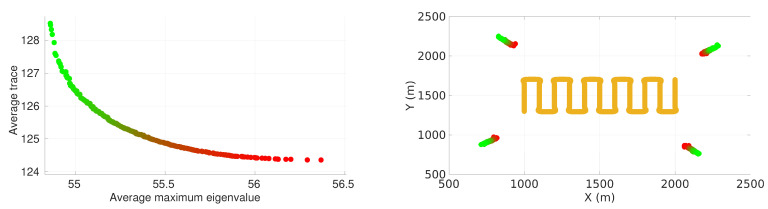
Pareto front considering average maximum eigenvalue and trace of inverse of FIM with respective positioning of sensors for the lawn-mower path.

In this section, we present the results for the multi-objective problem. The results allow us to analyze how the sensor positioning evolves when considering pair of metrics based on the FIM. We present the Pareto fronts for the lawn-mower maneuver with the four-sensor case. Figure 6 presents the front when considering the average determinant and the average maximum eigenvalue of the inverse of the FIM as pair of criteria. Figure 7 presents the front when considering the average trace and the average maximum eigenvalue of the inverse of the FIM as pair of criteria. In each figure, the sensor positioning is on the right and the corresponding value of the front is on the left.

It is possible to observe that in both cases the average maximum eigenvalue criteria tended to position the sensors closer to the vehicle trajectory compared to the other criteria. As in the single-objective case, the sensors of practically all solutions on both fronts were approximately the same distance from and equally distributed around the center of the target plane.

## 5. Conclusions

In the present work, we proposed a GA for the problem of optimum sensor placement for target localization with range-only measurements. Due to the nature of the algorithm, it can be applied in a wide range of cases, avoiding the necessity of derivation of solutions for specific scenarios, the typical approach in the marine applications literature. In this sense, the present algorithm could serve as a planning tool for missions that require the localization of underwater vehicles. We presented results for a single-objective problem, considering the average maximum eigenvalue of the FIM along the vehicle trajectory as the evaluation metric. Results were compared with a case with a known optimal solution, and simulations were performed considering range-dependent noises for a lawn-mower and a spiral descent maneuver. We also presented results for a multi-objective problem, considering pairs of criteria based on the FIM.

Algorithms based on heuristics and metaheuristics are powerful tools for complex optimization problems. However, the interpretation and the development of a method to generalize the results of specific cases to any case is not a trivial task, and perhaps, not possible. Nevertheless, more studies are necessary to better interpret the results of complex problems, such as the presented in this work, and to combine these with known analytical results for simpler cases to develop intuition and general methods for sensor placement in complex scenarios. Moreover, in this work we presented the placement of static sensors considering two very traditional maneuvers of autonomous underwater vehicles in separate scenarios. A future interesting study would be the consideration of moving sensors and the development of motion strategies for the sensors, for position reallocation between maneuvers while maintaining optimality.

## Figures and Tables

**Table 1:** Differences between our work and some of the relevant literature. The checkmarks represent the points covered by a particular paper.

Ref	Method		Criteria				Noise		Measurement				
	Analy	HM	D	A	E	Multi	PD	PI	R	RD	ToA	TDoA	AoA
Our work		✓			✓	✓	✓		✓				
[26]	✓	✓	✓					✓	✓				
[20]		✓	✓	✓	✓	✓	✓			✓			
[2]	✓		✓					✓			✓	✓	✓
[6]	✓		✓				✓		✓				
[7]	✓		✓					✓	✓		✓		
[16]		✓	✓										
[9]	✓			✓				✓			✓		

**Table 2:** Deviation from optimal solution and computational time.

# Sensors	Mean Deviation from Optimal (%)	Mean Computational Time (s)
4	0.073	7.43
5	0.056	8.11
6	0.071	8.67
7	0.039	9.28
8	0.045	9.28

**Table 3:** Solution with 4 sensors for the lawn-mower maneuver.

Sensor	X Position	Y Position	Z Position
1	712	2126	0
2	827	746	0
3	2283	871	0
4	2168	2253	0

**Table 4:** Distance and measurements standard deviation to the center of the place for the solution with 4 sensors and the lawn-mower maneuver.

Sensor	Distance to Center (m)	$\sigma_{c}$ (m)
1	1350	10.25
2	1353	10.28
3	1348	10.24
4	1350	10.25
mean	1350	10.25

**Table 5:** Results for lawn-mower trajectory.

# Sensors	Worst Axis of Uncertainty (m)	$\sigma_{c}$ (m)	Computational Time (s)
4	8.15	10.25	1521
5	7.08	10.30	1698
6	6.22	10.27	1804
7	5.76	10.25	1859
8	5.32	10.26	1977

**Table 6:** Solution with 4 sensors restricted to half plane for the lawn-mower maneuver.

Sensor	X Position	Y Position	Z Position
1	1989	1500	0
2	2030	2689	0
3	966	2693	0
4	996	1500	0

**Table 7:** Results for lawn-mower trajectory with sensors restricted to half of the plane.

# Sensors	Worst Axis of Uncertainty (m)	$\sigma_{c}$ (m)	Computational Time (s)
4	11.41	9.94	1740
5	10.02	10.55	1834
6	9.32	10.97	1746
7	8.52	10.53	1771
8	8.00	10.84	1929

**Table 8:** Solution with 4 sensors for the spiral descent maneuver.

Sensor	X Position	Y Position	Z Position
1	1624	2181	0
2	1947	1075	0
3	1580	1547	0
4	958	1334	0

**Table 9:** Results for spiral descent trajectory.

# Sensors	Worst Axis of Uncertainty (m)	Computational Time (s)
4	9.33	2390
5	7.53	2309
6	6.70	2575
7	611	2652
8	5.64	2875

**Figure A1:**
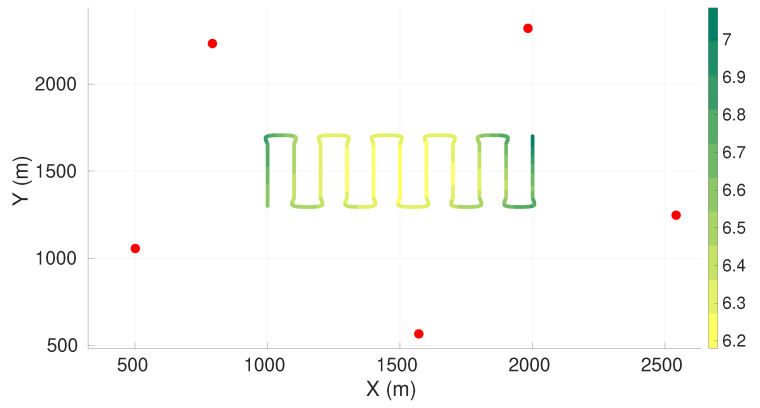
Maximum axis of uncertainty along vehicle trajectory for the lawn-mower maneuver and five sensors. Red dots represent the sensors, and the green gradient line represents the maximum axis of uncertainty along the vehicle trajectory.

**Figure A2:**
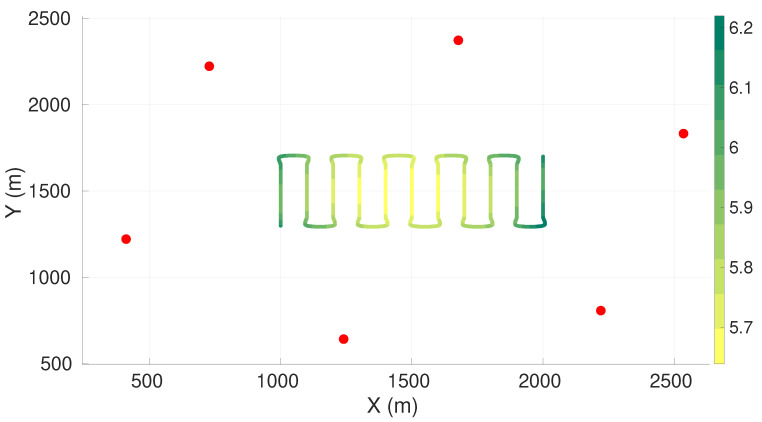
Maximum axis of uncertainty along vehicle trajectory for the lawn-mower maneuver and six sensors. Red dots represent the sensors, and the green gradient line represents the maximum axis of uncertainty along the vehicle trajectory.

**Figure A3:**
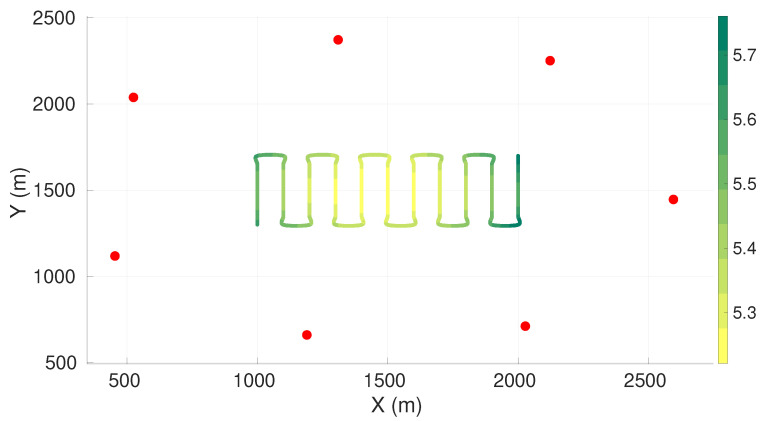
Maximum axis of uncertainty along vehicle trajectory for the lawn-mower maneuver and seven sensors. Red dots represent the sensors, and the green gradient line represents the maximum axis of uncertainty along the vehicle trajectory.

**Figure A4:**
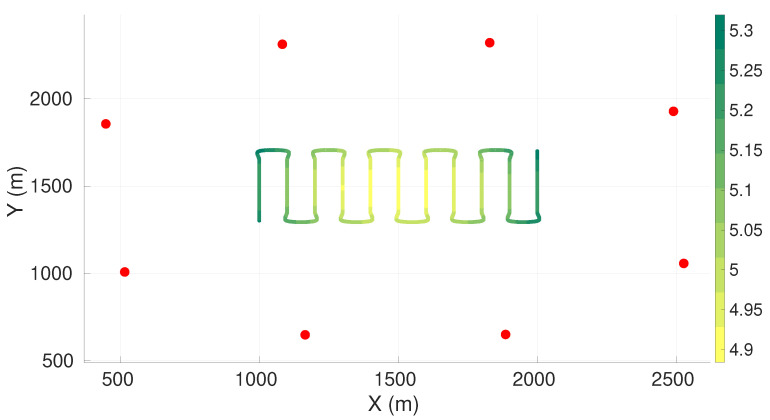
Maximum axis of uncertainty along vehicle trajectory for the lawn-mower maneuver and eight sensors. Red dots represent the sensors, and the green gradient line represents the maximum axis of uncertainty along the vehicle trajectory.

**Figure A5:**
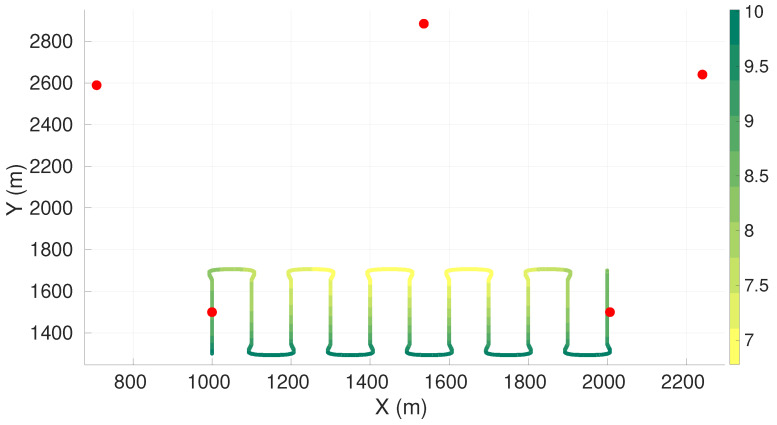
Maximum axis of uncertainty along vehicle trajectory for the lawn-mower maneuver and five sensors constrained to half of the plane. Red dots represent the sensors, and the green gradient line represents the maximum axis of uncertainty along the vehicle trajectory.

**Figure A6:**
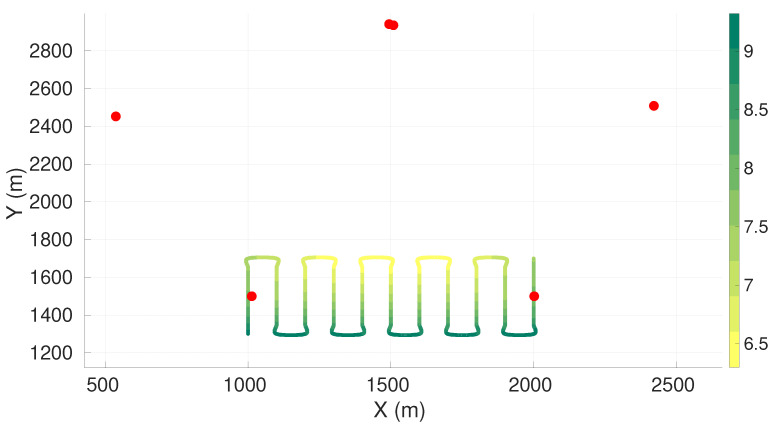
Maximum axis of uncertainty along vehicle trajectory for the lawn-mower maneuver and six sensors constrained to half of the plane. Red dots represent the sensors, and the green gradient line represents the maximum axis of uncertainty along the vehicle trajectory.

**Figure A7:**
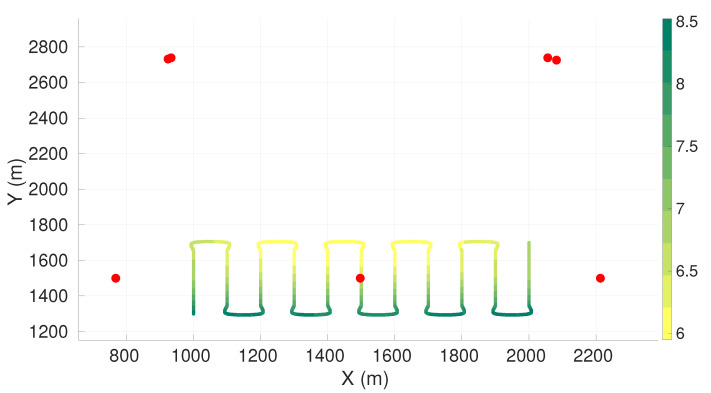
Maximum axis of uncertainty along vehicle trajectory for the lawn-mower maneuver and seven sensors constrained to half of the plane. Red dots represent the sensors, and the green gradient line represents the maximum axis of uncertainty along the vehicle trajectory.

**Figure A8:**
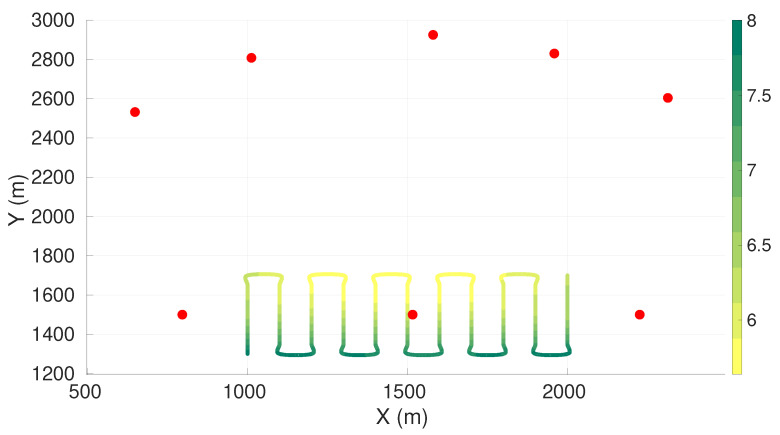
Maximum axis of uncertainty along vehicle trajectory for the lawn-mower maneuver and eight sensors constrained to half of the plane. Red dots represent the sensors, and the green gradient line represents the maximum axis of uncertainty along the vehicle trajectory.

**Figure A9:**
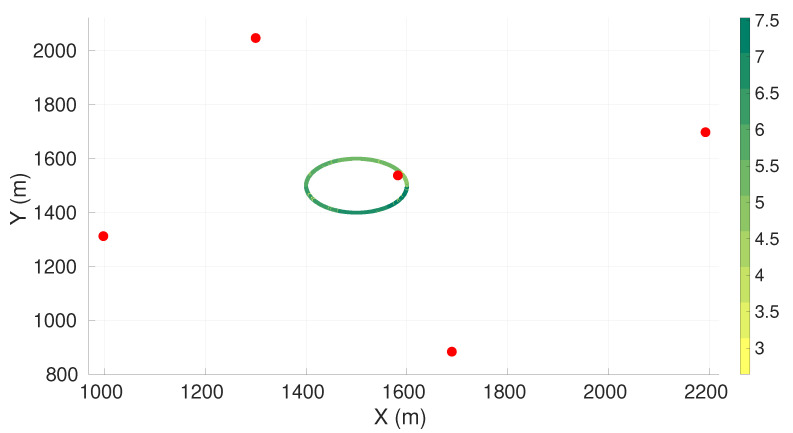
Maximum axis of uncertainty along vehicle trajectory for the spiral descent maneuver and five sensors. Red dots represent the sensors, and the green gradient line represents the maximum axis of uncertainty along the vehicle trajectory.

**Figure A10:**
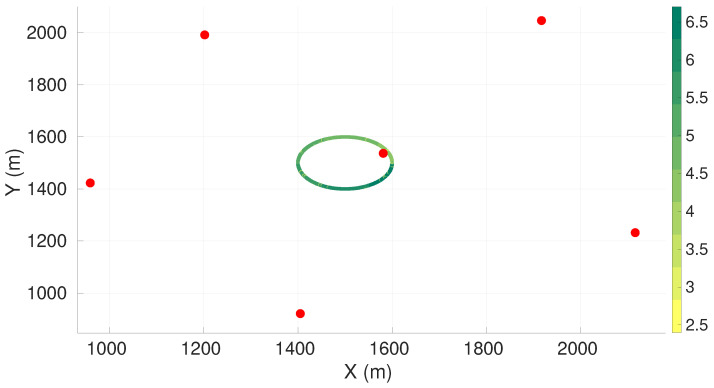
Maximum axis of uncertainty along vehicle trajectory for the spiral descent maneuver and six sensors. Red dots represent the sensors, and the green gradient line represents the maximum axis of uncertainty along the vehicle trajectory.

**Figure A11:**
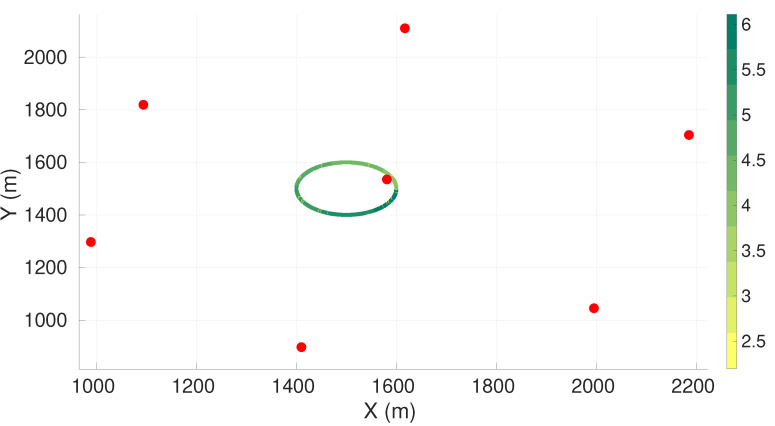
Maximum axis of uncertainty along vehicle trajectory for the spiral descent maneuver and seven sensors. Red dots represent the sensors, and the green gradient line represents the maximum axis of uncertainty along the vehicle trajectory.

**Figure A12:**
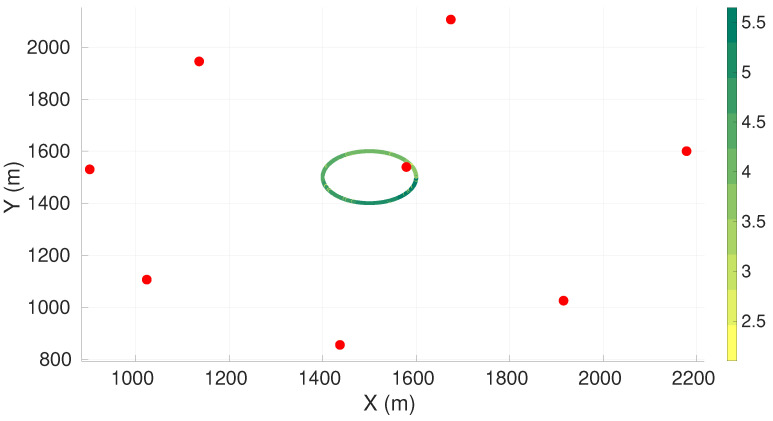
Maximum axis of uncertainty along vehicle trajectory for the spiral descent maneuver and eight sensors. Red dots represent the sensors, and the green gradient line represents the maximum axis of uncertainty along the vehicle trajectory.

## Data Availability

Not applicable.

## Appendix A. Tables of Sensor Positions

### Appendix A.1. Lawn-Mower

**Table A1:** Solution with 5 sensors for the lawn-mower maneuver.

Sensor	X Position	Y Position
1	792	2232
2	2542	1247
3	1571	566
4	1983	2319
5	501	1056

**Table A2:** Solution with 6 sensors for the lawn-mower maneuver.

Sensor	X Position	Y Position
1	2220	808
2	2535	1833
3	441	1222
4	1240	643
5	1677	2373
6	728	2223

**Table A3:** Solution with 7 sensors for the lawn-mower maneuver.

Sensor	X Position	Y Position
1	2123	2252
2	2596	1447
3	525	2039
4	2028	712
5	1190	661
6	1310	2373
7	454	1119

**Table A4:** Solution with 8 sensors for the lawn-mower maneuver.

Sensor	X Position	Y Position
1	448	1856
2	516	1008
3	2527	1057
4	1886	650
5	1165	648
6	1829	2321
7	1083	2312
8	2490	1928

### Appendix A.2. Lawn-Mower with Sensors in Half Plane

**Table A5:** Solution with 5 sensors in half plane for the lawn-mower maneuver.

Sensor	X Position	Y Position
1	708	2589
2	2007	1500
3	1000	1500
4	2241	2640
5	1536	2884

**Table A6:** Solution with 6 sensors in half plane for the lawn-mower maneuver.

Sensor	X Position	Y Position
1	2002	1500
2	537	2453
3	1494	2942
4	2421	2509
5	1509	2936
6	1013	1500

**Table A7:** Solution with 7 sensors in half plane for the lawn-mower maneuver.

Sensor	X Position	Y Position
1	924	2732
2	2056	2739
3	2213	1500
4	1497	1500
5	768	1500
6	2082	2726
7	933	2739

**Table A8:** Solution with 8 sensors in half plane for the lawn-mower maneuver.

Sensor	X Position	Y Position
1	1012	2808
2	648	2532
3	1580	2925
4	1516	1500
5	796	1500
6	2226	1500
7	1959	2830
8	2314	2604

### Appendix A.3. Spiral Descent

**Table A9:** Solution with 5 sensors for the spiral descent maneuver.

Sensor	X Position	Y Position
1	1689	885
2	2192	1698
3	998	1313
4	1582	1538
5	1300	2047

**Table A10:** Solution with 6 sensors for the spiral descent maneuver.

Sensor	X Position	Y Position
1	1405	921
2	1581	1537
3	1202	1992
4	2116	1232
5	959	1423
6	1917	2047

**Table A11:** Solution with 7 sensors for the spiral descent maneuver.

Sensor	X Position	Y Position
1	989	1297
2	1581	1535
3	1995	1045
4	2185	1704
5	1094	1819
6	1617	2110
7	1410	897

**Table A12:** Solution with 8 sensors for the spiral descent maneuver.

Sensor	X Position	Y Position
1	1437	855
2	1024	1106
3	1579	1539
4	902	1530
5	1674	2106
6	1915	1025
7	1136	1945
8	2178	1600

## References

[1] Wang Y., Ho K.C. (2013). TDOA Source Localization in the Presence of Synchronization Clock Bias and Sensor Position Errors. IEEE Trans. Signal Process..

[2] Bishop A.N., Fidan B., Anderson B.D., Doğançay K., Pathirana P.N. (2010). Optimality analysis of sensor-target localization geometries. Automatica.

[3] Moreno-Salinas D., Pascoal A., Aranda J. (2016). Optimal Sensor Placement for Acoustic Underwater Target Positioning with Range-Only Measurements. IEEE J. Ocean. Eng..

[4] Bishop A.N., Fidan B., Anderson B.D., Doǧançay K., Pathirana P.N. Optimality analysis of sensor-target geometries in passive localization: Part 1—Bearing-only localization. Proceedings of the 2007 International Conference on Intelligent Sensors, Sensor Networks and Information.

[5] Costa R. (2021). The Dependence of Aggregation in the Determination of Optimal Sensor Configurations for Source Localization. IEEE Trans. Aerosp. Electron. Syst..

[6] Fang X., Yan W., Chen W. (2016). Sensor Placement for Underwater Source Localization With Fixed Distances. IEEE Geosci. Remote Sens. Lett..

[7] Martínez S., Bullo F. (2006). Optimal sensor placement and motion coordination for target tracking. Automatica.

[8] Moreno-Salinas D., Pascoal A.M., Aranda J. (2013). Optimal sensor placement for multiple target positioning with range-only measurements in two-dimensional scenarios. Sensors.

[9] Xu S., Rice M., Rice F. (2021). Optimal TOA-Sensor Placement for Two Target Localization Simultaneously Using Shared Sensors. IEEE Commun. Lett..

[10] Bishop A.N., Fidan B., Anderson B.D., Pathirana P.N., Doǧançay K. Optimality analysis of sensor-target geometries in passive localization: Part 2—Time-of-arrival based localization. Proceedings of the 2007 International Conference on Intelligent Sensors, Sensor Networks and Information.

[11] Doğançay K., Hmam H. On optimal sensor placement for time-difference-of-arrival localization utilizing uncertainty minimization. Proceedings of the 2009 17th European Signal Processing Conference.

[12] Isaacs J.T., Klein D.J., Hespanha J.P. Optimal sensor placement for time difference of arrival localization. Proceedings of the 48h IEEE Conference on Decision and Control (CDC) held jointly with 2009 28th Chinese Control Conference.

[13] Neering J., Fischer C., Bordier M., Maizi N. Optimal sensor configuration for passive position estimation. Proceedings of the 2008 IEEE/ION Position, Location and Navigation Symposium.

[14] Sadeghi M., Behnia F., Amiri R. (2021). Optimal Geometry Analysis for TDOA-Based Localization under Communication Constraints. IEEE Trans. Aerosp. Electron. Syst..

[15] Yang B., Scheuing J. Cramer-Rao bound and optimum sensor array for source localization from time differences of arrival. Proceedings of the ICASSP, IEEE International Conference on Acoustics, Speech and Signal Processing.

[16] Yang Y., Zheng J., Liu H., Ho K., Chen Y., Yang Z. (2022). Optimal sensor placement for source tracking under synchronization offsets and sensor location errors with distance-dependent noises. Signal Process..

[17] Abel J.S. Optimal sensor placement for passive source localization. Proceedings of the ICASSP, IEEE International Conference on Acoustics, Speech and Signal Processing.

[18] Bo X., Razzaqi A.A., Wang X., Farid G. (2020). Optimal geometric configuration of sensors for received signal strength based cooperative localization of submerged AUVs. Ocean. Eng..

[19] Moreno-Salinas D., Pascoal A., Aranda J. (2011). Optimal Sensor Placement for Multiple Underwater Target Localization with Acoustic Range Measurements. IFAC Proc. Vol..

[20] Domingo-Perez F., Lazaro-Galilea J.L., Wieser A., Martin-Gorostiza E., Salido-Monzu D., de la Llana A. (2016). Sensor placement determination for range-difference positioning using evolutionary multi-objective optimization. Expert Syst. Appl..

[21] Sahu N., Wu L., Babu P., MR B.S., Ottersten B. (2022). Optimal Sensor Placement for Source Localization: A Unified ADMM Approach. IEEE Trans. Veh. Technol..

[22] Levanon N. (2000). Lowest GDOP in 2-D scenarios. IEE Proc.-Radar Sonar Navig..

[23] Yoon Y., Kim Y.H. (2013). An Efficient Genetic Algorithm for Maximum Coverage Deployment in Wireless Sensor Networks. IEEE Trans. Cybern..

[24] Chen C.S., Chiu Y.J., Lee C.T., Lin J.M. (2013). Calculation of Weighted Geometric Dilution of Precision. J. Appl. Math..

[25] Du Z., Wang W., Chai H., Xiang M., Zhang F., Huang Z. (2022). Configuration analysis method and geometric interpretation of UUVs cooperative localization based on error ellipse. Ocean. Eng..

[26] Moreno-Salinas D., Pascoal A.M., Aranda J. (2018). Multiple underwater target positioning with optimally placed acoustic surface sensor networks. Res. Artic. Int. J. Distrib. Sens. Netw..

[27] Xu S., Wu L., Dogancay K., Alaee-Kerahroodi M. (2022). A Hybrid Approach to Optimal TOA-Sensor Placement With Fixed Shared Sensors for Simultaneous Multi-Target Localization. IEEE Trans. Signal Process..

[28] Díez-González J., Verde P., Ferrero-Guillén R., Álvarez R., Pérez H. (2020). Hybrid Memetic Algorithm for the Node Location Problem in Local Positioning Systems. Sensors.

[29] Ferrero-Guillén R., Díez-González J., Álvarez R., Pérez H. (2020). Analysis of the Genetic Algorithm Operators for the Node Location Problem in Local Positioning Systems. Proceedings of the International Conference on Hybrid Artificial Intelligence Systems.

[30] Moreno-Salinas D., Pascoal A., Aranda J. (2013). Sensor Networks for Optimal Target Localization with Bearings-Only Measurements in Constrained Three-Dimensional Scenarios. Sensors.

[31] Ferreira B.M., Matos A.C., Campos H.S., Cruz N.A. Localization of a sound source: Optimal positioning of sensors carried on autonomous surface vehicles. Proceedings of the 2013 OCEANS.

[32] Díez-González J., Álvarez R., González-Bárcena D., Sánchez-González L., Castejón-Limas M., Perez H. (2019). Genetic Algorithm Approach to the 3D Node Localization in TDOA Systems. Sensors.

[33] Díez-González J., Álvarez R., Verde P., Ferrero-Guillén R., Perez H. (2022). Analysis of reliable deployment of TDOA local positioning architectures. Neurocomputing.

[34] Chen B., Pandey P., Pompili D. (2012). An Adaptive Sampling Solution using Autonomous Underwater Vehicles. IFAC Proc. Vol..

[35] Bovio E., Cecchi D., Baralli F. (2006). Autonomous underwater vehicles for scientific and naval operations. Annu. Rev. Control.

[36] Lakhekar G.V., Waghmare L.M. (2017). Robust maneuvering of autonomous underwater vehicle: An adaptive fuzzy PI sliding mode control. Intell. Serv. Robot..

[37] Andonian M., Cazzaro D., Invernizzi L., Chyba M., Grammatico S. Geometric control for autonomous underwater vehicles: Overcoming a thruster failure. Proceedings of the 49th IEEE Conference on Decision and Control (CDC).

[38] Yan W., Fang X., Li J. (2014). Formation Optimization for AUV Localization With Range-Dependent Measurements Noise. IEEE Commun. Lett..

[39] Weiss A., Picard J. (2008). Network Localization with Biased Range Measurements. IEEE Trans. Wirel. Commun..

[40] Kay S.M. (1993). Fundamentals of Statistical Signal Processing: Estimation Theory.

[41] Konak A., Coit D.W., Smith A.E. (2006). Multi-objective optimization using genetic algorithms: A tutorial. Reliab. Eng. Syst. Saf..

[42] Zitzler E., Thiele L. (1999). Multiobjective evolutionary algorithms: A comparative case study and the strength Pareto approach. IEEE Trans. Evol. Comput..

[43] Deb K., Pratap A., Agarwal S., Meyarivan T. (2002). A Fast and Elitist Multiobjective Genetic Algorithm: NSGA-II. IEEE Trans. Evol. Comput..

